# Comparison of Treatment Outcome between Hypofractionated Radiotherapy and Conventional Radiotherapy in Postmastectomy Breast Cancer

**DOI:** 10.31557/APJCP.2020.21.1.119

**Published:** 2020

**Authors:** Chokaew Tovanabutra, Kanyarat Katanyoo, Pichet Uber, Kittisak Chomprasert, Sitti Sookauychai

**Affiliations:** 1 *Radiation Oncology section, *; 3 *Medical Oncology Section, Chonburi Cancer Hospital, Chonburi, *; 2 *Department of Radiology, Faculty of Medicine, Vajira Hospital, Navamindradhiraj University, Bangkok, Thailand. *

**Keywords:** Hypofractionated radiotherapy, conventional radiotherapy, post mastectomy breast cancer

## Abstract

**Objective:**

The aim of this study was to compare Conventional fractionated radiotherapy (CFRT) and Hypofractionated radiotherapy (HFRT) in terms of treatment outcomes, such as in 5-year loco-regional recurrence free survival, disease free survival, overall survival, and distant metastatic free survival rates as well as toxicity.

**Materials and Methods:**

We retrospectively analyzed the data obtained from 462 breast cancer patients who received complete adjuvant radiotherapy treatment between January 2012 and December 2014. One hundred twenty eight patients received CFRT 2 Gy daily fractions at a total dose of 48-60 Gy (group 1), while 334 patients received HFRT 2.65-2.67 Gy daily for 15-19 fractions at a total dose of 39.7-47.8 Gy 9 (grup 2). Treatment outcome such as 5-year loco-regional recurrence free survival, disease free survival, overall survival, and distant metastatic free survival rates as well as toxicity were measured and compared between two groups.

**Results:**

Median follow-up was 65.7 months (ranging from 45.1 to 95.2 months). Five-year loco-regional recurrence free survival rate was 96.1% in both CFRT and HFRT groups (p=0.993). Five-year disease-free survival rate of CFRT group was higher (68.8%), but this difference was not significant (HFRT =63.5%) (p=0.396). These were complied with 5-year overall survival rate (71.9% and 64.7%, p=0.385). Five-year distant metastatic free survival rate was 85.9% in CFRT group and 79.6% in HFRT group (p=0.169). No difference was observed between two groups in terms of toxicities, including changes in chest wall appearance, skin fib rosis, brachial plexopathy, arm edema, pulmonary fibrosis, and cardiovascular events.

**Conclusion:**

The treatment outcomes of hypofractionated radiotherapy in the postmastectomy breast cancer patients is comparable to the outcomes of conventional treatment at the Chonburi Cancer Hospital as previously reported from other studies, and HFRT can be implemented in resource-limited settings.

## Introduction

Breast cancer is the most common cancers among Thai women. Treatment of locally advanced breast cancer includes surgery, chemotherapy and/or targeted therapy, hormonal therapy, and radiotherapy. The patients who have mastectomy and cancer spread to the axillary lymph nodes or large tumor size need adjuvant radiotherapy in order to reduce recurrence rate and increase their survival (Ragaz et al., 1997; Nielsen et al., 2006; McGale et al., 2014) 

Conventional adjuvant radiotherapy for postmastectomy chest wall is giving 2-Gy daily fractions over 5-6 weeks (CFRT). The radiobiological model on hypofractionated radiotherapy (HFRT), which reduces the number of fractions and overall treatment time by using larger doses > 2 Gy per fraction, was reported to be as effective as the conventional longer schedule (Fowler, 1989). The results from a large randomized trials on HFRT in women undergoing whole breast irradiation after breast conserving surgery showed similar efficacy and late effects as conventionally fractionated radiotherapy (Whelan et al., 2010; Agrawal et al., 2011; Haviland et al., 2013). In addition, HFRT has been used in post mastectomy patients who require adjuvant radiotherapy, especially in the countries with limited radiotherapy resources, in order to decrease patient waiting time to start radiotherapy and increase patient turnover rate. There were many centers reported similar efficacy and toxicity of HFRT to CFRT (Pinitpatcharalert et al., 2011; Eldeeb et al., 2012; Alam et al., 2016; Wang et al., 2019).

Under these circumstances, the Chonburi Cancer Hospital has introduced HFRT to treat patients with post mastectomy breast cancers since 2012. In the current investigation, CFRT and HFRT were compared in terms of treatment outcomes, such as 5-year loco-regional recurrence free survival, disease free survival, overall survival, and distant metastatic free survival rates as well as toxicity .

## Materials and Methods

This retrospective study was performed on 462 post mastectomy breast cancer patients who received complete adjuvant radiation therapy at Chonburi Cancer Hospital between January 2012 and December 2014. This study was approved by the Ethical Committee of Chonburi Cancer Hospital. Informed consent was obtained from each patient. 

Inclusion criteria were as follows: being female, suffering from with breast cancer stage II or III, according to 7^th^ edition of AJCC Cancer Staging, and having a histologically proven invasive carcinoma (Edge and Compton, 2010). Exclusion criteria were having surgical margin of mastectomy specimen less than 1 mm, immediate reconstruction after mastectomy, incomplete medical record, or history of systemic lupus erythematosus, scleroderma, or previous breast/chest irradiation. 

Patient demographic and clinicopathological data including age, cancer staging, histopathology, hormonal status, chemotherapy, hormonal treatment, radiotherapy field, and dose were collected. Chemotherapy regimens included antracyclin-based, taxane-based, and CMF. Hormonal treatment involved tamoxifen and aromatase inhibitors. 

*Radiotherapy protocol*

All patients were treated on a SIEMENS MEVATRON MX2 or VARIAN CLINAC CX 2300C linear accelerator 6MV machine. Patients were treated with the supine position with breast board and arm support . All of them were simulated with 2D technique. The target volume included the chest wall with or without axillary and supraclavicular lymph nodes. Radiotherapy was given using two tangential fields as well as anteroposterior supraclavicular fields. A bolus was used over the chest wall field daily for the first half of radiotherapy course. Anterior supraclavicular field was delivered to patients with any number of positive dissected axillary lymph nodes, and some cases of N0 with the presence of lymphovascular invasion and tumor grade 3. Axillary boost was allowed in case of clinical N2 disease, inadequate node excision (less than 10 nodes), or extracapsular extension. The patients were irradiated with 2 schedules: conventional radiotherapy (CFRT), 48.0-60.0 Gy (2.0 Gy in 24-30 fractions), and hypofractionated radiotherapy (HFRT), 39.7-47.8 Gy (2.65-2.67 Gy in 15-19 fractions). However, due to the long waiting period for radiation in the Chonburi Cancer Hospital, most of the patients have been delivered HFRT since the end of 2012.

*Endpoints*

The endpoints of this study included loco-regional recurrence free survival (LRFS), disease-free survival (DFS), overall survival (OS), and distant metastatic free survival (DMFS). LRFS was defined as time from the date of surgical treatment to in-field radiotherapy recurrence. DFS was defined as time from the date of surgical treatment to any breast cancer related events (loco-regional recurrence and distant metastasis) or death. OS was defined as time from the date of surgical treatment to death from any reasons . DMFS was defined as time from the date of surgical treatment to any distant metastatic events. In addition, acute and late toxicity grading was performed according to RTOG/EORTC radiation morbidity scoring criteria (Cox et al., 1995). The Chonburi Cancer Hospital’s Lymphedema score, developed by the department of surgery since 2010 based on Harris et al., (2001), was used for evaluating lymphedema. Arm circumference was taken at the middle of dorsum of hand, at the wrist level, and 10 cm above and below the lateral epicondyles and was compared with the opposite arm. The highest difference during follow up was taken as the final measurement. The grading scale used for scoring lymphedema was as follows: grade 0 (no difference), grade 1 (increase less than 2 cm), grade 2 (increase of 2-5 cm), and grade 3 (increase more than 5 cm). All patients were assessed acute/late toxicities weekly during radiotherapy and 6 weeks after the end of radiotherapy and then were followed up every 3-6 months in person or by phone. 

*Statistical Methods*

Data were analyzed by using SPSS (version 22) (SPSS Inc., Chicago, IL). Descriptive statistic was used to analyze clinicopathological and treatment data, which were demonstrated as number and percentage. The homogeneity of the studied population was evaluated by Chi-square test. Five-year loco-regional recurrence free survival (LRFS), disease-free survival (DFS), overall survival (OS), and distant metastatic free survival (DMFS) rates were studied by using Kaplan-Meier analysis. To compare two groups, log rank test was run. The Cox proportional hazards model was applied to adjust all important prognostic factors. The backward elimination method was used for selecting significant prognostic factors with p-to-remove > 0.10. A two-sided P-value < 0.05 was considered statistically significance. 

## Results

*Patient characteristics*

A total of 462 breast cancer patients underwent post-mastectomy radiation therapy during January 2012 and December 2014 were recruited in this study, of whom 128 (27.7%) underwent CFRT and 334 (72.3%) underwent HFRT. The mean age of patients was 50.6 **±** 10.0 years old. Table 1 shows the demographic and clinical characteristics of the patients. The HFRT group had significantly higher rate of T4 (10.8% vs 3.9%, p=0.030) and of N3 (22.2% vs 11.7%, p=0.022) when compared to the CFRT group. There were no significant differences between two groups in terms of age, cancer staging, histopathology, tumor grading, lymphovascular invasion, extracapsular nodal extension, ER, PR, HER2, type of chemotherapy, chemotherapy regimen, and hormonal treatment. 

*Disease’s status at the last follow-up*

Median follow-up duration was 65.7 months (range, 45 months to 95 months). At the time of analysis, 168 patients (36.4%) died, of whom one hundred and twenty eight patients (27.7%) died of cancer (5.6% in CFRT and 22.1% in HFRT), 17 patients (3.7%) died from non-cardiac causes (1.7% in CFRT and 2% in HFRT), and 23 patients (5%) died from unknown causes (2% in CFRT and 3% in HFRT).

**Figure 1:**
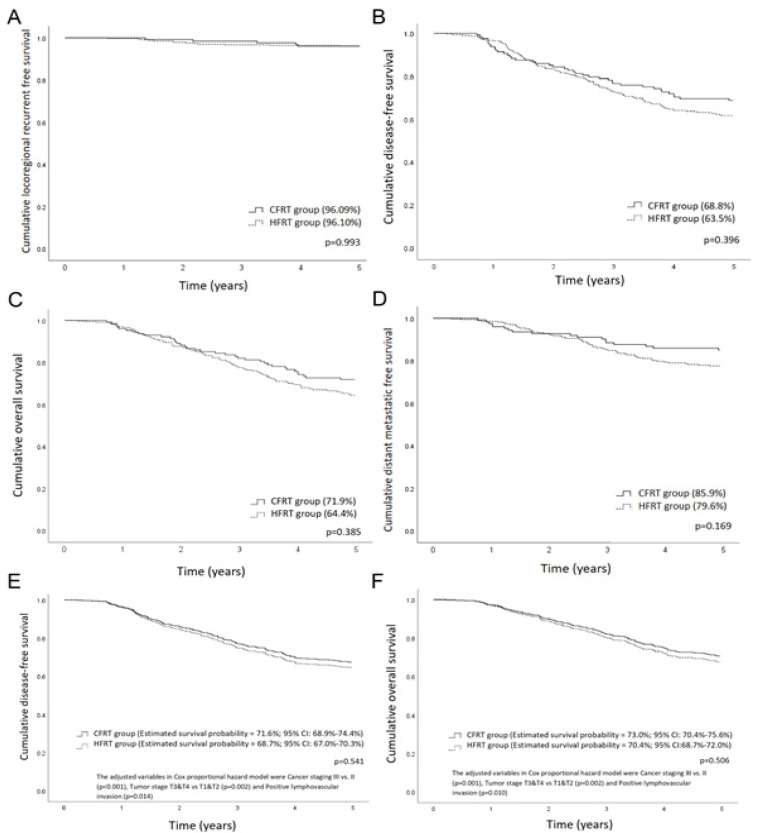
Loco-Regional Recurrence Free Survival Rate (A); Disease-free survival rate (B); Overall survival rate (C); Distant metastatic free survival rate (D); Adjusted disease-free survival rate (E); Adjusted overall survival rate (F).

There was no statistical difference between both groups regarding the 5-year LRFS. The estimated cumulative incidences of 5-year loco-regional recurrence using the Kaplan–Meier approach was 3.89% (95% CI, 1.81-5.98) in the HFRT group and was 3.91% (95% CI, 0.50-7.31) in the CFRT group. The 5-year LRFS was comparable in CFRT and HFRT groups (Figure 1A). However, the 5-year survival (DFS, OS) was higher in the CFRT group than the HFRT group, but it was not statistically significant (p=0.396 and p=0.385, respectively) (Figures 1B and 1C). Out of 462 patients, distant metastasis occurred in 90 patients (19.48%), and the most common site was bone (34.44%), followed by lung (27.78%) and brain (21.11%). The 5-year DMFS was also higher, as DFS and OS, in the CFRT group when compared to HFRT group, but it was not statistically significant either (p=0.169) (Figure 1D).

The univariate analysis was performed on 5-year follow up and it was revealed that cancer stage III, tumor stage 3-4, nodal stage 2-3, and lymphovascular invasion were significantly unfavorable prognostic factors for overall survival (Table 2). A trend of being unfavorable prognostic factor in HFRT was also seen, but it was not statistically significant. However, multivariate analysis finally indicated that cancer stage III, tumor stage 3-4, nodal stage 2-3, and lymphovascular invasion had 1.9-, 1.6-, and 1.4-fold hazard ratio for death, respectively. Meanwhile, HFRT did not affect the overall survival of the patients. In addition, the adjusted survival curve, based on Cox proportional hazard models, did not show any differences between two groups regarding the estimated disease free survival probability (71.6% in CFRT and 68.7% in HFRT, p=0.541) and overall survival (73.0% in CFRT and 70.4% in HFRT, p=0.506) (Figures 1E and 1F).

*Treatment toxicities*

Table 3 presents the different grades of acute and late toxicity. The incidence of grade 2 acute dermatitis was different, 42% in CFRT group and 5.7% in HFRT group. Merely one patient in HFRT group had grade 1 acute pulmonary toxicity. Among late toxicities diagnosed, higher grades of subcutaneous tissue fibrosis and arm edema were found in both groups. Subcutaneous tissue fibrosis grade 2 in CFRT group was found at 0.8% while grades 2 and 3 were found at 1.2% and 0.3% in HFRT group. Arm edema grade 2 was found 3.3% in CFRT group while grades 2 and 3 were found at 1.9% and 0.6% in HFRT group. Grade 3 arm edema in HFRT-arm was found in patients received axilla radiation. Encouragingly, there was no report on grade 4 subcutaneous tissue fibrosis and arm edema in HFRT group. Most clinically diagnosed pulmonary toxicity was grade 0, and only 11.4% and 9.3% of grade 1 were reported in CFRT and HFRT groups. Meanwhile, chest radiography identified grade 1 in CFRT, and HFRT group at 58.3% and 46.6%, respectively. There was no cardiotoxicity and severe brachial plexopathy in both groups.

**Table 1:** Characteristics of Patients in This Study (N=462)

Characteristics	CFRT (N=128)	HFRT (N=334)	P value
Age (years)			0.89
<40	17 (13.3)	46 (13.8)	
40 or more	111 (86.7)	288 (86.3)	
Cancer staging			0.087
II**	51 (39.8)	105 (31.4)	
III**	77 (60.2)	229 (68.6)	
Histopathology			0.628
Invasive ductal	121 (94.5)	322 (96.4)	
Invasive lobular	3 (2.3)	6 (1.8)	
Others	4 (3.2)	6 (1.8)	
Grade			0.683
1	7 (5.5)	16 (4.8)	
2	58 (45.3)	163 (48.8)	
3	53 (41.4)	138 (41.3)	
Unknown	10 (7.8)	17 (5.1)	
Side			0.481
Right	62 (48%)	174 (52%)	
Left	66 (52%)	160 (48%)	
Tumor stage			0.030*
T1	6 (4.7)	28 (8.4)	
T2	78 (60.9)	194 (58.1)	
T3	39 (30.5)	76 (22.8)	
T4	5 (3.9)	36 (10.8)	
Nodal stage			0.022*
N0	18 (14.1)	53 (15.9)	
N1	50 (39.1)	91 (27.2)	
N2	45 (35.2)	116 (34.7)	
N3	15 (11.7)	74 (22.2)	
Number of node dissection			0.453
0-9	25 (19.5)	76 (22.8)	
10 or more	103 (80.5)	258 (77.2)	
Lymphovascular invasion			0.217
Negative	85 (66.4)	201 (60.2)	
Positive	43 (33.6)	133 (39.8)	
Extracapsular nodal extension			0.465
Negative	114 (89.1)	289 (86.5)	
Positive	14 (10.9)	45 (13.5)	
ER			0.146
Negative	60 (46.9)	131 (39.2)	
Positive	66 (51.5)	188 (56.3)	
Unknown	2 (1.6)	15 (4.5)	
PR			0.193
Negative	78 (60.9)	183 (54.8)	
Positive	48 (37.5)	135 (40.4)	
Unknown	2 (1.6)	16 (4.8)	
HER2			0.608
Negative (0/+1/+2)	88 (68.7)	217 (65.0)	
Positive (+3)	34 (26.6)	94 (28.1)	
Unknown	6 (4.7)	23 (6.9)	
Characteristics	CFRT (N=128)	HFRT (N=334)	P value
Type of chemotherapy			0.054
None	1 (0.8)	1 (0.3)	
Neoadjuvant	2 (1.6)	26 (7.8)	
Adjuvant	111 (86.7)	262 (78.4)	
Neoadjuvant + Adjuvant	14 (10.9)	45 (13.5)	
Chemotherapy regimen			0.672
None	1 (0.8)	1 (0.3)	
Antracyclin based	40 (31.3)	107 (32.0)	
Taxane based	81 (63.3)	218 (65.3)	
CMF	1 (0.8)	2 (0.6)	
Unknown	5 (3.9)	6 (1.8)	
Hormonal treatment			0.704
None	63 (49.2)	146 (43.7)	
Tamoxifen	47 (36.7)	142 (42.5)	
Aromatase inhibitors	10 (7.8)	20 (6.0)	
Tamoxifen + Aromatase inhibitors	3 (2.3)	9 (2.7)	
Unknown	5 (3.9)	17 (5.1)	
Axilla Irradiation	32 (25.0%)	60 (18%)	0.09

*, Homogeneity test by Chi-square, two-sided P-value<0.05; **, For patients who did not receive neoadjuvant chemotherapy, we used pathological stage because it was more accurate than clinical stage. For patients who received neoadjuvant chemotherapy, we used whichever stage was higher (clinical or pathological) to reflect the actual tumor burden; ER, Estrogen receptor; HER 2, Human epidermal growth factor receptor 2; N, Regional lymph nodes; PR, Progesterone receptor; T, Primary tumor.

**Table 2:** Univariate and Multivariate Analysis of Correlated to Overall Survival

Variables	Univariate		Multivariate†	
	HR (95% CI)	p-value	HR (95% CI)	p-value
RT regimen				
CFRT	1		1	
HRFT	1.318 (0.908 - 1.913)	0.147	-	
Age				
<40	1		1	
40 or more	0.795 (0.533 - 1.184)	0.258	-	
Cancer staging				
II	1		1	
III	2.304 (1.561 - 3.400)	<0.001	1.925 (1.283 – 2.890)	0.002
Tumor stage				
T1 and T2	1		1	
T3 and T4	1.782 (1.298 – 2.446)	<0.001	1.620 (1.164 – 2.255)	0.004
Nodal stage				
N0 and N1	1		1	
N2 and N3	1.719 (1.237 – 2.389)	0.001	-	
Lymphovascular invasion				
Negative	1		1	
Positive	1.439 (1.049 – 1.974)	0.024	1.396 (1.008 – 1.932)	0.045
Extracapsular nodal extension				
Negative	1		1	
Positive	0.954 (0.590 – 1.541)	0.847	-	

†, The variables were excluded from the equation when p-to-remove was greater than 0.10; HFRT, regiment (p=0.196); Age >= 40 years (p=0.302), Nodal stage N2 and N3 (p=0.370) and positive of Extracapsular nodal extension (p=0.317)

**Table 3:** Toxicities According to the Fractionation Schedule

Treatment toxicities	CFRT (n=128) (%)				HFRT (n=334) (%)			
	Grade 0	Grade 1	Grade 2	Grade 3-4	Grade 0	Grade 1	Grade 2	Grade 3-4
Acute								
Dermatitis	30 (23.4)	44 (34.4)	54 (42.2)	0	222 (66.5)	93 (27.8)	19 (5.7)	0
Pneumonitis	128 (100)	0	0	0	333 (99.7)	1 (0.3)	0	0
Late*								
Skin atrophy	51 (41.5)	71 (57.7)	1 (0.8)	0	121 (37.6)	197 (61.2)	4 (1.2)	0
Subcutaneous tissue fibrosis	57 (46.3)	65 (52.9)	1 (0.8)	0	135 (42)	182 (56.5)	4 (1.2)	1 (0.3)
Angina/Pericarditis	123 (100)	0	0	0	322 (100)	0	0	0
Esophageal stricture	123 (100)	0	0	0	320 (99.4)	2 (0.6)	0	0
Shoulder stiffness	118 (95.9)	5 (4.1)	0	0	303 (94.1)	18 (5.6)	1 (0.3)	0
Arm edema	118 (95.9)	1 (0.8)	4 (3.3)	0	305 (94.7)	9 (2.8)	6 (1.9)	2 (0.6)
Lung fibrosis (Clinical)	109 (88.6)	14 (11.4)	0	0	292 (90.7)	30 (9.3)	0	0
Lung fibrosis** (Chest film)	10 (41.7)	14 (58.3)	0	0	31 (53.4)	27 (46.6)	0	0

All other data are presented as the number (%) of patients; *There were 17 patients in both arms died before 6 months, therefore acute toxicity couldn’t be evaluate in 5 patients (3.9%) in CFRT group and 12 patients (3.6%) in HFRT group; **Chest film has been evaluated for late toxicity in 82 patients, 24 patients (19.5%) in CFRT group and 58 patients (18%) in HFRT group respectively.

## Discussion

The Chonburi cancer hospital is the only healthcare center that provides radiotherapy services in eastern part of Thailand. With growing numbers of cancer patients every year and the limited resources, CFRT has been replaced by HFRT in post mastectomy breast cancer. This study was conducted to compare the results of the two treatment groups. The results revealed that the 5-year loco-regional recurrence free survivals of both methods were similar at 96.1% (p=0.093). The 5- year disease-free survival of CFRT group (68.8%) was higher than that of HFRT group (63.5%), but this difference was not statistically different (p=0.396). The same trend was also seen for overall survival in CFRT (71.9%) and HFRT (64.4%) groups (p=0.385). 

The estimated cumulative incidences of 5-year loco-regional recurrence in the HFRT and CFRT groups were not different in a study conducted by Wang et al., (2019). In our study, the 5-year loco-regional recurrence was also similar between two groups, 3.89% (95% CI, 1.81-5.98) in HFRT group and 3.91% (95% CI,0.50-7.3) in CFRT group; however, the values were lower than those reported by Wang et al., (2019), ( 8.3% (90% CI 5.8-10.7) in HFRT group and 8.1% (90% CI 5.4-10.6) in CFRT group (absolute difference 0.2%, 90% CI -3.0 to 2.6; HR 1.10, 90% CI 0.72 to 1.69). These lower local recurrence differences can be due to fewer numbers of patients at stage III (68%) in our study when compared number of these patients in aforementioned study (94%). The 5-year DFS was 63.5% in HRFT group and 68.8% in CFRT group, revealing no significant difference. Similar to our findings, in a study conducted by Pinitpatcharalert et al., (2011), 5-year DFS in two groups was not different, that is 62.7% in CFRT group and 69.6% in HFRT group (p=0.136). These findings were in line with a similar study by Wang et al., (2019), who performed a randomized phase III study on high risk group with postmastectomy breast cancer. They reported 74% in HFRT group and 70% in CFRT group (p=0.42).

Pinitpatcharalert et al., (2011) showed that the difference between CFRT and HFRT was significantly higher in patients of hypofractionated group in terms of 5-year overall survival (62.7% vs. 73.0%) (p=0.048). In contrast to study of Wang et al. (2019) which revealed 5-yr overall survival were comparable in both groups, 86 % in CFRT and 84% in HFRT (p=0.526). Meanwhile, the 5-year overall survival in our study was lower in HFRT group (64.4%) compared to CFRT group (71.9%), but this difference was not statistically significant. 

Considering the lower DFS and OS in HFRT group when compared to CFRT group, although not significant, these differences likely due to the limitation of retrospective study. Clinical and demographic characteristics of patients were not optimum randomized. Data collection was not completed due to follow-up of some patients who were referred to other hospitals. Results of multivariate analysis revealed that cancer stage III, tumor stage 3-4, and lymphovascular invasion significantly determined the poor OS; whereas, HFRT did not have an effect on OS outcome. In addition, the adjusted survival curve showed no significant difference in both groups regarding estimated survival probability. Therefore, the lower overall survival in HFRT group of our study caused by higher numbers of patients with cancer stage III and tumor stage 4 comparing to CFRT group. 

Acute dermatitis is the most common radiation-induced toxicity. Only grade 2 toxicities or more were concerned because most patients were expected to have at least grade 1 skin toxicity. Rastogi et al., (2018) found grade 2 acute dermatitis 40% in CFRT and 42% in HFRT. Similarly, by Alam et al., (2016) that grade 2 were seen at 42% in CFRT and 54% in HFRT. Kouloulias et al., (2016) reported grade 2-3 dermatitis at 29%, while Ko et al., (2015) reported grade 2 at 10.7% in HFRT. The present study found grade 2 at 42.2% in CFRT which is comparable to the results from Rastogi et al., (2018) and Alam et al., (2016). 

Cardiotoxicity is a late complication which significantly occurs in patients who receive left-sided radiotherapy (Sardar et al., 2017). The risk of coronary heart disease usually starts within the first decade, while cardiac mortality might appear in the second decade (Cheng et al., 2017). However, no significant difference was reported between patients undergoing CFRT or HFRT regarding cardiovascular mortality rate (Chan et al., 2015). In this study, we also found no patients with cardiac complication that could be due to short follow-up duration (65.7 months). The other reason can be due to this fact that neither EKG nor echocardiography was performed to detect the cardiac complication after post radiation. Hence, there might be some cardiovascular events from radiation and have never been documented. There were 23 patients who deceased with unknown caused that might be the cardiovascular event(s) from radiation. 

*Brachial plexopathy was experienced in HFRT group*

According to Galecki et al., (2006), the use of doses per fraction in the range of 2.2 Gy and 4.58 Gy with the total doses between 43.5 Gy and 60 Gy significantly increased the risk of brachial plexus injury from 1.7% up to 73%. The risk of radiation induced brachial plexopathy was smaller than 1% after administrating of doses per fraction between 2.2 Gy and 2.5 Gy with the total dose between 34 Gy and 40 Gy. Khan et al., (2017) found clinically symptomatic/significant brachial plexopathy 12.5% in post mastectomy patients received hypofractionated schedules either 42.5 Gy in 16 fractions with 2.65 Gy fraction. In Pinitpatcharalet et al.,’s study (2011), the incidence rate of brachial plexopathy was low and comparable with that of conventional regimen because the radiation fraction size was less than 3 Gy and the total dose was also reduced. In the present study, no clinical or symptomatic brachial plexopathy were found in both groups, which is in accordant with a previous study done by Kouloulias et al., (2016) revealing no plexopathy in any of the groups (HFRT 48.30 Gy in 21 fractions, 42.56 Gy in 16 fractions, and CFRT 50 Gy in 25 fractions).

For other grade 3 late toxicities, subcutaneous tissue fibrosis and arm edema were less than 1% in HFRT group. However, the grade 3 of arm edema was only found in patients who received posterior axillary boost; therefore, the axilla irradiation might be limited in case of clinical N2 or pathological extracapsular nodal extension in order to decrease this toxicity. Grade 0 to 1 of lung fibrosis, joint stiffness and esophageal stricture were identified, but it led to no significant differences between two groups. 

Some of the limitations of this study are related to its design, the imbalance of two treatment arms, and the use of two dimension radiation techniques. This study has some advantages such as considering long follow up period compared to other studies and collecting data only one center so the radiation techniques were uniform. 

In conclusion, the treatment outcomes following HFRT in the post mastectomy breast cancer patients were comparable to those following CFRT at Chonburi Cancer Hospital, which is in line with the findings of previous studies. However, the authors suggested the use of the 3D conformal radiation which is currently and widely used instead of 2D radiation to improve dose homogeneity and minimize the needed dose for normal organs. These results will give the confidence in the standard of practice and cares to the medical personnel and patients at this hospital. The implementation of findings of this study can also lead to treatment cost reduction due to fewer numbers of fractions and hospital visits. 

## References

[B1] Agrawal RK, Alhasso A, Barrett-Lee PJ (2011). First results of the randomised UK FAST Trial of radiotherapy hypofractionation for treatment of early breast cancer (CRUKE/04/015). Radiother Oncol.

[B2] Alam MS, Perween R, Siddiqui AS (2016). Accelerated hypofrationated radiation in carcinoma breast. Arch Cancer Res.

[B3] Chan EK, Woods R, Virani S (2015). Long-term mortality from cardiac causes after adjuvant hypofractionated vs conventional radiotherapy for localized left-sided breast cancer. Radiother Oncol.

[B4] Cheng YJ, Nie XY, Ji CC (2017). Long-term cardiovascular risk after radiotherapy in women with breast cancer. J Am Heart Assoc.

[B5] Cox JD, Stetz J, Pajak TF (1995). Toxicity criteria of the Radiation Therapy Oncology Group (RTOG) and the European Organization for Research and Treatment of Cancer (EORTC). Int J Radiat Oncol Biol Phys.

[B6] Edge SB, Compton CC (2010). The American Joint Committee on Cancer: the 7th Edition of the AJCC Cancer Staging Manual and the Future of TNM. Ann Surg Oncol.

[B7] Eldeeb H, Awad I, Elhanafy O (2012). Hypofractionation in post-mastectomy breast cancer patients: seven-year follow-up. Med Oncol.

[B8] Fowler JF (1989). The linear-quadratic formula and progress in fractionated radiotherapy. Br J Radiol.

[B9] Galecki J, Hicer-Grzenkowicz J, Grudzien-Kowalska M (2006). Radiation-induced brachial plexopathy and hypofractionated regimens in adjuvant irradiation of patients with breast cancer--a review. Acta Oncol.

[B10] Harris SR, Hugi MR, Olivotto IA, Levine M (2001). Clinical practice guidelines for the care and treatment of breast cancer: 11. Lymphedema. CMAJ.

[B11] Haviland JS, Owen JR, Dewar JA (2013). The UK Standardisation of Breast Radiotherapy (START) trials of radiotherapy hypofractionation for treatment of early breast cancer: 10-year follow-up results of two randomised controlled trials. Lancet Oncol.

[B12] Jahanzeb M (2008). Adjuvant trastuzumab therapy for HER2-positive breast cancer. Clin Breast Cancer.

[B13] Khan M, Siddiqui SA, Gupta MK (2017). Normal tissue complications following hypofractionated chest wall radiotherapy in breast cancer patients and their correlation with patient, tumor, and treatment characteristics. Indian J Med Paediatr Oncol.

[B14] Ko DH, Norriss A, Harrington CR (2015). Hypofractionated radiation treatment following mastectomy in early breast cancer: the Christchurch experience. J Med Imaging Radiat Oncol.

[B15] Kouloulias V, Mosa E, Zygogianni A (2016). A retrospective analysis of toxicity and efficacy for 2 hypofractionated irradiation schedules versus a conventional one for post-mastectomy adjuvant radiotherapy in breast cancer. Breast Care (Basel).

[B16] McGale P, Taylor C, Correa C (2014). Effect of radiotherapy after mastectomy and axillary surgery on 10-year recurrence and 20-year breast cancer mortality: meta-analysis of individual patient data for 8135 women in 22 randomised trials. Lancet.

[B17] Nielsen HM, Overgaard M, Grau C (2006). Study of failure pattern among high-risk breast cancer patients with or without postmastectomy radiotherapy in addition to adjuvant systemic therapy: long-term results from the Danish Breast Cancer Cooperative Group DBCG 82 b and c randomized studies. J Clin Oncol.

[B18] Pinitpatcharalert A, Chitapanarux I, Euathrongchit J (2011). A retrospective study comparing hypofractionated radiotherapy and conventional radiotherapy in postmastectomy breast cancer. J Med Assoc Thai.

[B19] Ragaz J, Jackson SM, Le N (1997). Adjuvant radiotherapy and chemotherapy in node-positive premenopausal women with breast cancer. N Engl J Med.

[B20] Rastogi K, Jain S, Bhatnagar AR (2018). A comparative study of hypofractionated and conventional radiotherapy in postmastectomy breast cancer patients. Asia Pac J Oncol Nurs.

[B21] Sardar P, Kundu A, Chatterjee S (2017). Long-term cardiovascular mortality after radiotherapy for breast cancer: A systematic review and meta-analysis. Clin Cardiol.

[B22] Wang SL, Fang H, Song YW (2019). Hypofractionated versus conventional fractionated postmastectomy radiotherapy for patients with high-risk breast cancer: a randomised, non-inferiority, open-label, phase 3 trial. Lancet Oncol.

[B23] Whelan TJ, Pignol JP, Levine MN (2010). Long-term results of hypofractionated radiation therapy for breast cancer. N Engl J Med.

